# Strain Identity of the Ectomycorrhizal Fungus *Laccaria bicolor* Is More Important than Richness in Regulating Plant and Fungal Performance under Nutrient Rich Conditions

**DOI:** 10.3389/fmicb.2017.01874

**Published:** 2017-09-26

**Authors:** Christina Hazard, Laura Kruitbos, Hazel Davidson, Fatou T. Mbow, Andy F. S. Taylor, David Johnson

**Affiliations:** ^1^Environmental Microbial Genomics, École Centrale de Lyon, Université de Lyon, Ecully, France; ^2^Institute of Biological and Environmental Sciences, University of Aberdeen, Aberdeen, United Kingdom; ^3^The James Hutton Institute, Aberdeen, United Kingdom; ^4^School of Earth and Environmental Sciences, University of Manchester, Manchester, United Kingdom

**Keywords:** diversity, ectomycorrhizal fungi, ecosystem function, *Laccaria bicolor*, niche partitioning, nitrogen, nutrients, productivity

## Abstract

Effects of biodiversity on productivity are more likely to be expressed when there is greater potential for niche complementarity. In soil, chemically complex pools of nutrient resources should provide more opportunities for niche complementarity than chemically simple pools. Ectomycorrhizal (ECM) fungal genotypes can exhibit substantial variation in nutrient acquisition traits and are key components of soil biodiversity. Here, we tested the hypothesis that increasing the chemical complexity and forms of soil nutrients would enhance the effects of intraspecific ECM diversity on host plant and fungal productivity. In pure culture, we found substantial variation in growth of strains of the ECM fungus *Laccaria bicolor* on a range of inorganic and organic forms of nutrients. Subsequent experiments examined the effects of intraspecific identity and richness using Scots pine (*Pinus sylvestris*) seedlings colonized with different strains of *L. bicolor* growing on substrates supplemented with either inorganic or organic forms of nitrogen and phosphorus. Intraspecific identity effects on plant productivity were only found under the inorganic nutrient amendment, whereas intraspecific identity affected fungal productivity to a similar extent under both nutrient treatments. Overall, there were no significant effects of intraspecific richness on plant and fungal productivity. Our findings suggest soil nutrient composition does not interact strongly with ECM intraspecific richness, at least under experimental conditions where mineral nutrients were not limiting. Under these conditions, intraspecific identity of ECM fungi becomes more important than richness in modulating plant and fungal performance.

## Introduction

Research on biodiversity-ecosystem function relationships has tended to focus on species diversity (Loreau et al., 2001; Tilman et al., 2014) with much less emphasis on within-species diversity (Hughes et al., 2008). Increasingly, however, intraspecific diversity has been shown to have wide-ranging effects on numerous ecosystem processes in a myriad of systems (Hughes et al., 2008; Johnson et al., 2012). Yet, little is known about the nature of the biodiversity-ecosystem function relationship with respect to forest ecosystems and intraspecific diversity of soil microorganisms. A key group of forest soil microorganisms are the ectomycorrhizal (ECM) fungi, which compose a significant proportion of the soil microbial biomass (Högberg and Högberg, 2002), and colonize the majority of the fine root systems of trees in boreal and temperate biomes (Smith and Read, 2008). Ectomycorrhizal fungi have important functional roles in forest ecosystems, particularly for biogeochemical cycling and nutrient acquisition to trees (Smith and Read, 2008). Molecular analyses of ECM fungal communities demonstrate considerable variation both within and between species, at a range of spatial scales (Johnson et al., 2012). Indeed, the roots of an individual *Populus tremula* tree were found to support between 182 and 207 species of ECM fungi, and 23 ITS genotypes of *Cenococcum geophilum* (Bahram et al., 2010).

Recent findings indicate that intraspecific diversity of ECM fungi can have significant impacts on ecosystem processes and multifunctionality (Wilkinson et al., 2010, 2012; Hazard et al., 2017). For example, intraspecific diversity of *Paxillus obscurosporus* was found to affect fungal productivity and respiration in pure culture (Wilkinson et al., 2010). A more challenging set of experiments found that intraspecific diversity of *Laccaria bicolor* affected productivity of host plants, fungal productivity and soil nutrient retention, and that the effect of richness on these metrics were stronger at the intra- versus interspecific level of diversity (Hazard et al., 2017). These studies suggest that intraspecific identity and richness of ECM fungi can play a direct role in influencing forest ecosystem functioning, and complementarity, derived from niche differentiation and facilitation, is likely the explanatory mechanism (Johnson et al., 2012). This is supported by the finding that *L. bicolor* populations overyield when in mixture compared to what would be predicted from their performance in monoculture (Hazard et al., 2017).

While these studies highlight the need to consider the role of intraspecific diversity in regulating ecosystem functioning, a major gap in understanding concerns the conditions in which such effects are likely to occur. Positive, negative or neutral effects of ECM intraspecific diversity on ecosystem functioning metrics, such as plant productivity, are likely context dependent, as seen in studies manipulating interspecific richness. For example, the biomass of birch plants, originally associated with eight ECM species, was doubled in comparison to single ECM associations but only when soil fertility was low (Jonsson et al., 2001). Thus, there is a need to conduct experiments under various abiotic environmental conditions to increase predictability power of the impact ECM diversity could have on forest ecosystem functioning.

The productivity of boreal and temperate forests is contingent on nitrogen (N) availability: these systems are typically low in available N (LeBauer and Treseder, 2008; Inselsbacher and Näsholm, 2012). Characteristically, ECM plants dominate these forests, where their associated ECM fungi possess the ability to scavenge for essential nutrients from the soil, in particular N and phosphorus (P), and transfer a portion of these nutrients to their tree hosts in exchange for reduced carbon (Smith and Read, 2008). Boreal and temperate forest soils contain both inorganic and organic sources of N and P, although organic forms are found in much greater quantities (Inselsbacher and Näsholm, 2012; Bol et al., 2016). ECM fungi have evolved to utilize both inorganic forms and low molecular weight organic forms of N such as amino acids, which can be taken-up intact (e.g., Näsholm et al., 1998), and ECM fungi can also grow on organic forms of P such as phytic acid (Antibus et al., 1992). Differences in nutrient complexities in soil may be an important mechanism by which niche separation and substrate use preferences occur that would reduce competition between individuals. Resource heterogeneity in soils may also be a key selective force leading to differences in functional traits of ECM fungi, given that resource heterogeneity is an important factor that supports the biodiversity of grassland and animal communities (Kerr and Packer, 1997; Harpole and Tilman, 2007).

Evidence from culture studies has shown that species of ECM fungi differ in their utilization of N sources (Abuzinadah and Read, 1988; Finlay et al., 1992; Anderson et al., 1999), and even between individuals within a species (Finlay et al., 1992; Anderson et al., 1999; Sawyer et al., 2003; Guidot et al., 2005). It is likely that strains of ECM fungi also differ in the ability to utilize organic P forms, as evident by variation in the production of organic P degrading enzymes (Ho, 1989; Meyselle et al., 1991; Antibus et al., 1992). For example, strains of *L. bicolor* have been shown to vary in their solubilization of inorganic phosphorus sources in culture (Nguyen et al., 1992; de la Bastide et al., 1995). It is therefore plausible that due to differences in N and P use efficiencies between strains within a species that ECM intraspecific diversity could affect components of the N cycle in forest ecosystems. Also, as host plant growth can be contingent on the ECM strain (Hedh et al., 2009), intraspecific diversity could have a key role in tree productivity. Indeed, in Hazard et al. (2017), *L. bicolor* intraspecific diversity was found to have considerable effects on the productivity of both fungi and Scots pine hosts in microcosms. Their experimental design consisted of a gradient of intraspecific richness, with each strain in monoculture and in mixture to enable testing for both intraspecific identity and richness effects, and transgressive overyielding (Loreau, 1998; Loreau and Hector, 2001). Here we use the same *L. bicolor* strains and experimental design, but with the growth medium enriched with either inorganic or organic forms of N and P nutrient resources. An *a priori* pure culture experiment showed the ability of the *L. bicolor* strains to differentially utilize forms of inorganic and organic N, and showed greater variation in utilization for organic versus inorganic N, and suggesting the potential for nutrient partitioning. Here, we test for direct effects of *L. bicolor* intraspecific diversity on Scots pine and fungal productivity, and determine whether these effects are driven by interactions among fungal strains and between strains and nutrient complexity. We predict that the effects of *L. bicolor* intraspecific identity and richness on Scots pine plant and fungal productivity will be greatest when the chemical composition and variety of nutrient resources is complex, due to the greater likelihood of niche partitioning.

## Materials and Methods

### Strains of *Laccaria bicolor*

Four strains of *Laccaria bicolor* (Marie) P. D. Orton (LbA, LbB, LbC, LbD), described in Hazard et al. (2017), were used for experiments. From an ecological perspective, this ECM species was chosen because it is a common early-stage colonizer of Scots pine, and other tree hosts in boreal and temperate forests (Richter and Bruhn, 1989; Buschena et al., 1992). Also, individuals of *L. bicolor* have been shown to persist within a population for at least 3 years (de la Bastide et al., 1994). The *L. bicolor* strains used in this study were all derived from Scots pine forests on podzol soils within an area of 916 km^2^ in Aberdeenshire, Scotland. These strains have relatively considerable genetic variation between them. Comparison between the strains’ internal transcribed spacer (ITS) region of rDNA, a conserved genetic marker for fungal species, shows overall sequence variability ranging from 97% to 99.4% (NCBI accession numbers: MF958447-MF958450). Furthermore, genomic comparison between the strains and single nucleotide polymorphisms (SNP) analyses based on key functional genes (e.g., transporter genes for amino acids and ammonia) supports a large potential for variation in functional attributes between these strains (unpublished data; C. Hazard).

### An *A priori* Pure Culture Experiment

The four strains of *L. bicolor* (LbA, LbB, LbC, LbD) were tested for their ability to utilize the different inorganic and organic nitrogen sources for growth in pure culture. Sterile 9 cm Petri dishes were filled with 25 ml of a Modified Melin Norkins (MMN, Marx, 1969) solution that excluded malt extract and contained 0.03 g l^-1^ of N either in the form of ammonium nitrate, glutamic acid, glycine, phenylalanine, or no nitrogen source. Glucose was added to the MMN solutions such that the final C:N ratio was 20:1, and the pH of the solutions were adjusted to 5.5 with 1M NaOH. A sterile cellophane disk (Type 325P) (Cannins Packaging Ltd., Bristol, United Kingdom) was placed on top of the agar media, and an 8 mm disk of mycelium taken from the growing edge of a 4-week old *L. bicolor* culture on MMN was placed on the cellophane. Four replicates for each *L. bicolor* strain by nitrogen source and harvest time were prepared, and incubated in the dark at 22°C for either 14 or 28 days. Mycelia was removed from the cellophane disks and dried in an oven at 80°C for 18 h and then weighed.

### Microcosms

Scots pine (*Pinus sylvestris* L.) seedlings individually colonized by one of four *L. bicolor* strains (LbA, LbB, LbC, LbD) under aseptic conditions were used to establish experimental microcosms. The methods for mycorrhizal seedling synthesis are detailed in Hazard et al. (2017). Individual pine seedlings were intentionally colonized with only one strain to avoid any confounding effects that could arise from unequal inoculum dispersal on seedling root systems (Leake, 2001). Briefly, sterile Scots pine seedlings (lot 08RP20SI, Forestry Commission, United Kingdom) were introduced onto *L. bicolor* culture plates [1.0 g l^-1^ glucose Modified Melin-Norkrans (MMN) agar overlaid with cellophane] and grown under controlled conditions (16 h light at 180 mmol photons m^-2^ s^-1^/8 h dark cycle, and constant temperature of 18°C) for 2 months. Seedling biomass, root length, and total number of *L. bicolor* root-tips were measured prior to planting into microcosms to account for any growth differences between the seedlings (Supplementary Table 1).

Microcosms were constructed in 17 cm × 12 cm plastic pots (LBS Worldwide Ltd., Colne, United Kingdom) filled with 500 g of homogenized, sterile (2x autoclaved at 121°C for 1 h) sand, vermiculite (Vermiculite V3 medium 2.0 – 5.0 mm; William Sinclair Horticulture Ltd., United Kingdom), and either an inorganic or organic N and P nutrient solution at the ratio of 5:1:3 respectively. The sand-vermiculite medium was chosen due to its negligible N (0.054 mg Total N g^-1^; 0.002 mg NH_4_-N g^-1^) and P (0.099 mg Total P g^-1^; 0.037 mg PO_4_-P kg^-1^) content, in comparison to that of other mediums. The nutrient solutions consisted of double strength low carbon (1.0 g l^-1^ glucose) MMN without malt extract and 0.061 g l^-1^ of N and 0.36 g l^-1^ of P. The total amount of N and P was the same in the inorganic and organic nutrient solutions, resulting in 10 mg of N and 60 mg of P added to each microcosm, providing a nutrient rich condition. The inorganic nutrient solution contained ammonium nitrate (0.175 g l^-1^) and potassium dihydrogen phosphate (1.6 g l^-1^) as the sole N and P source, respectively. The organic nutrient solution contained four amino acids, glycine (0.082 g l^-1^), glutamine (0.078 g l^-1^), glutamic acid (0.161 g l^-1^) and phenylalanine (0.181 g l^-1^) as the sole source of N, and phytic acid dipotassium salt (1.44 g l^-1^) as the sole P source. The N and P sources were chosen because these nutrient sources are found in forest soils (Inselsbacher and Näsholm, 2012; Bol et al., 2016), and ECM fungi, and specifically *L. bicolor*, have been shown to utilize these forms of N and P (Abuzinadah and Read, 1988; Finlay et al., 1992; Nguyen et al., 1992; Martin et al., 1994).

### Experimental Design

Colonized Scots pine seedlings were planted into the inorganic and organic substrate nutrient microcosms such that an intraspecific richness gradient was created, with each isolate in monoculture, in mixture of two strains and all four strains (**Table 1**). Each pot contained four seedlings, and each seedling was randomly designated a position within the pot, with the positions at equal distance from each other. Each identity treatment within each nutrient treatment was replicated six times resulting in 384 seedlings (8 identity treatments × 2 nutrient treatments × 6 replicates × 4 seedlings per pot). Non-mycorrhizal pine seedlings were used both as a test for overall effects of colonization and as a control to quantify for fungal contamination, of which none was detected. The microcosms were randomly placed within a growth chamber and incubated under controlled conditions (same as for mycorrhizal seedling synthesis) and watered with dH_2_O when required.

**Table 1:** Experimental design consisting of *Laccaria bicolor* (Lb) intraspecific monoculture and mixture treatments under inorganic and organic nutrient substrate across a richness gradient.

Fungal strains		Richness
Inorganic nutrient substrate	Organic nutrient substrate	
LbA	LbA	1
LbB	LbB	1
LbC	LbC	1
LbD	LbD	1
LbA + LbB	LbA + LbB	2
LbC + LbD	LbC + LbD	2
LbA + LbD	LbA + LbD	2
LbA + LbB + LbC + LbD	LbA + LbB + LbC + LbD	4

### Plant and Fungal Productivity Measurements

Microcosms were destructively harvested after 5 months to measure seedling shoot height, shoot biomass, shoot P, shoot N, root biomass, root length and ECM root-tips per root length (ECM-RRL). At harvest, seedling shoots were measured with a millimeter ruler before oven drying at 60°C for 48 h and weighing. Dried shoots were finely milled (Mixer Mill MM 400, Retsch, Haan, Germany; Smith et al., 2013) and digested in sulphuric acid:hydrogen peroxide (Allen, 1989), and the P and N concentrations were measured on a flow injection analyser (FIAstar 5000 system with 5027 Sampler; Foss NIRSytem Inc., Hillerød, Denmark). Seedling root systems were carefully washed in water, and positioned into clear trays with the roots immersed in water, and scanned (Epson Expression 10000XL; Epson Canada Ltd, Toronto, ON, Canada) to determine root length and number of root-tips using WinRHIZO Pro v2009 (Regent Instruments Canada Inc., Quebec, QC, Canada), and root biomass determined by weighing after oven drying. For each seedling, % ECM colonization was determined, and this value was multiplied by the number of root-tips and divided by root length (from root scanning) to calculate ECM-RRL.

### Data Analyses

To test for significant variation in fungal biomass between the *L. bicolor* strains that were grown on the different sources of N in pure culture, two-way analysis of variance was performed for each harvest time followed by *post hoc* Tukey’s tests.

Plant and fungal productivity measures were totaled across the four seedlings in a microcosm, and totaled values were used untransformed in statistical models. Overall main and interactive effects for nutrient complexity and intraspecific richness on the productivity variables were tested using linear mixed-effects models with intraspecific identity included as a random nested factor to account for the variance within the richness levels. Two-sample *t*-tests were used to determine if the productivity variables were significantly different between the nutrient treatments for each of the intraspecific identities. For each nutrient treatment, significant intraspecific identity effects on the productivity variables were tested using one-way analysis of variance and assuming unequal variances. When intraspecific identity effects were significant, *post hoc* Tukey’s tests were used to determine significant differences between the identities. Intraspecific richness effects on the productivity variables, for each nutrient treatment, were tested using linear mixed-effects models with intraspecific identity included as a random factor. Linear regression was used to test whether there was an increasing or decreasing trend with richness. Assessment of the seedling growth measures taken prior to planting revealed no significant differences between the identity treatments for each nutrient treatment (Supplementary Table 1), and thus were not considered in the above statistical models as a confounding source of error. Separate statistical analyses were conducted to test the effect of the extent of mycorrhizal colonization on the productivity variables (Supplementary Table 2), and therefore the non-mycorrhizal control was not included in the above statistical models. Statistical analyses were carried out in R v.3.3.0 with packages ‘nlme’ (ver. 3.1-131) for mixed-effects models and ‘stats’ (ver. 3.3.3) for the remaining analyses (R Core Team, 2016).

To determine if *L. bicolor* mixtures produced more (*D*_max_ > 0) or less (*D*_max_ < 0) than its most productive corresponding monoculture, transgressive overyielding was calculated [*D*_max_ = (observed yield of mixture) – (observed yield of maximally yielding monoculture)/(observed yield of maximally yielding monoculture)] (Loreau, 1998). One-sample *t*-tests were used to test the significance of *D*_max_ values from zero.

## Results

### Utilization of Nitrogen Sources by *L. bicolor* Strains in Pure Culture

**FIGURE 1:**
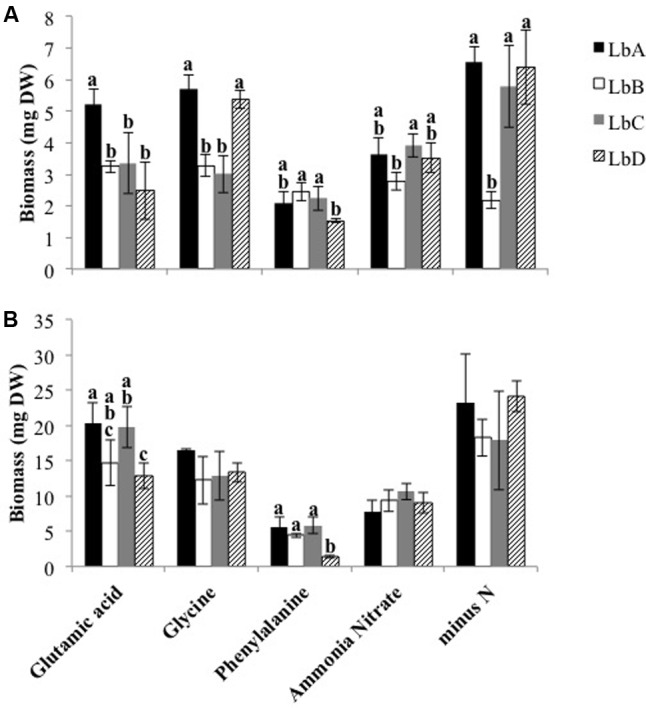
Biomass of *Laccaria bicolor* strains after **(A)** 14- and **(B)** 28-days of growth in pure culture with different nitrogen sources and without nitrogen (minus N). Based on Tukey’s *post hoc* test, bars with different letters indicate a significant difference in the mean biomass (±SD, *n* = 4) between strains for a nitrogen source.

The pure culture experiment demonstrated that the *L. bicolor* strains in this study utilized both inorganic and organic N sources (**Figure 1**), and that there was variation in growth between strains and the different N sources after 14 (*P*-value < 0.001, *F*-value = 10.94, strain^∗^N source) and 28 days (*P*-value = 0.010, *F*-value = 2.50, strain^∗^N source). For both harvest times, mean biomass was significantly different between all of the N sources (*P*-values < 0.009, 14 days; *P*-values < 0.046, 28 days), with the exception of the contrast difference between ammonium nitrate and glutamic acid after 14 days (*P*-value = 0.979). Strains growing on phenylalanine produced the least fungal biomass in comparison to the other N sources (*P*-values < 0.001), and LbD had significantly less biomass in comparison to the other strains (**Figure 1**). After 14 days, variation in biomass between some of the strains was found for each of the N sources (**Figure 1**). After 28 days, growth on glutamic acid and glycine was greater in comparison to ammonium nitrate (*P*-value < 0.001), and LbD had significantly less biomass in comparison to LbA and LbC (**Figure 1**). Fungal biomass of the strains was greater when grown without N, with the exception of LbB after 14 days (**Figure 1**).

### Effects of Nutrient Treatment and Richness on Productivity

**FIGURE 2:**
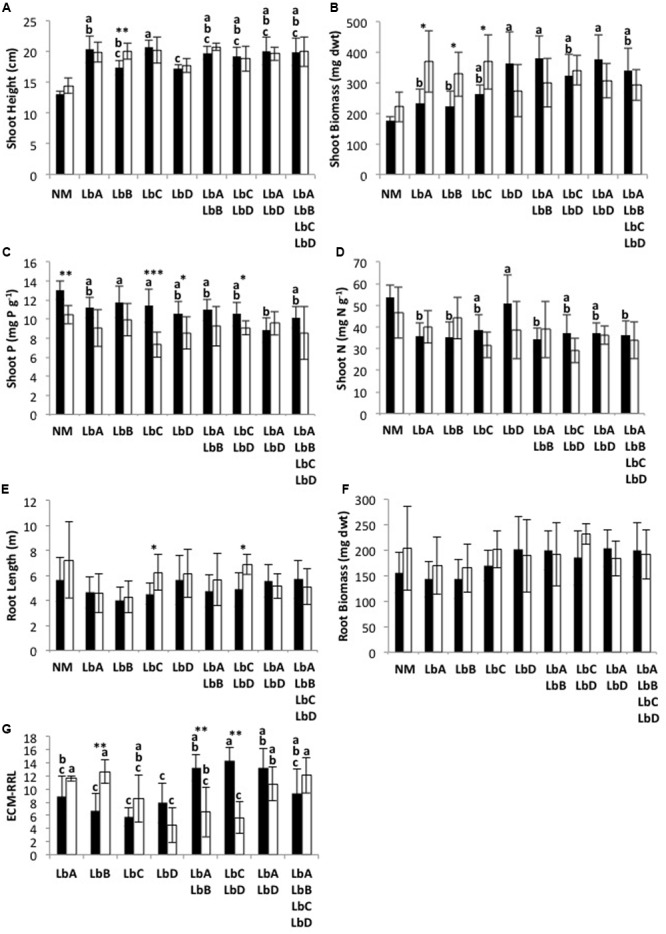
*Laccaria bicolor* intraspecific identity (LbA, LbB, LbC, LbD) effects on Scots pine **(A–F)** plant and **(G)** fungal productivity (ECM-RRL, ECM Root-tips per Root Length) under inorganic (black) and organic (white) nutrient substrates across a richness gradient. Treatment means (±SD) with different letters are significantly different based on Tukey’s *post hoc* test following one-way analysis of variance (NM, non-mycorrhizal control; not included in analysis), and significance levels for differences between nutrient treatments for each identity treatment are indicated as: ^∗^*P* < 0.05, ^∗∗^*P* < 0.01, ^∗∗∗^*P* < 0.001.

Nutrient treatment main effects were only significant for Scots pine shoot phosphorus content (*T* = -2.937; *P* = 0.013), with the Scots pine in the inorganic nutrient substrate having the greatest mean shoot phosphorus content (Supplementary Table 3). There was no *L. bicolor* intraspecific richness main effects (*P* > 0.263) or interaction effects between nutrient treatment and richness (*P* > 0.054) for any of the productivity variables (Supplementary Table 3). Although not significant (*T* = -2.147; *P* = 0.054), the Scots pine in the organic nutrient substrate resulted in the lowest mean shoot biomasses when in monoculture with *L. bicolor* but had the highest mean shoot biomasses when in mixture whereas the opposite trend was found for the inorganic nutrient substrate (**Figure 2B**).

### Variability in Productivity between Nutrient Treatments Due to Intraspecific Identity

Shoot height, shoot biomass, shoot P content, root length and ECM-RRL were all significantly affected by the nutrient treatment for at least one of the *L. bicolor* intraspecific identities. Shoot N content (*P* > 0.084) and root biomass (*P* > 0.093) were unaffected (**Figure 2**). Mean shoot height, shoot biomass and root length of Scots pine were significantly greater under the organic nutrient substrate for some of the *L. bicolor* strains (LbB for shoot height; LbB, LbA and LbC for shoot biomass; LbC and LbC + LbD for root length) (**Figures 2A–C**). In contrast, mean shoot P content of Scots pine was significantly greater under the inorganic nutrient substrate for the LbC, LbD and LbC + LbD identity treatments (**Figure 2C**). The monoculture LbB had significantly greater ECM-RRL under the organic nutrient substrate, whereas the mixtures LbA + LbB and LbC + LbD had significantly greater ECM-RRL under the inorganic nutrient substrate (**Figure 2G**).

### Intraspecific Identity Effects on Productivity within Nutrient Treatments

Intraspecific identity effects on Scots pine productivity were only found under the inorganic nutrient substrate. Shoot height (*F* = 3.91; *P* = 0.002), shoot biomass (*F* = 5.21; *P* < 0.001), shoot P content (*F* = 2.74; *P* = 0.020) and shoot N content (*F* = 2.79; *P* = 0.019) were significantly different between some of the *L. bicolor* identity treatments (**Figure 2**). Scots pine colonized by *L. bicolor* monocultures produced least biomass (**Figures 2A,B**), whereas mean shoot P content was generally greater in the monocultures than the mixtures (**Figure 2C**). The monoculture LbD had significantly greater mean shoot N content than most of the other *L. bicolor* monoculture and mixtures (**Figure 2D**). No significant *L. bicolor* identity effects on Scots pine root length and root biomass were found (**Figures 2E,F**).

Unlike plant productivity, significant effects of *L. bicolor* intraspecific identity were found on fungal productivity, and ECM root-tips per root length (ECM-RRL) under both inorganic (*F* = 8.66; *P* < 0.001) and organic (*F* = 8.37; *P* < 0.001) nutrient substrates (**Figure 2G**). For inorganic nutrient substrate, the *L. bicolor* monocultures had the least ECM-RRL and were significantly different from the two-strain mixtures, with the exception of LbA (**Figure 2G**). For organic nutrient substrate, the monoculture LbD had the least ECM-RRL and LbB had the greatest. The identit*y* treatments LbA, LbB, LbA + LbD and LbA + LbB + LbC + LbD had significantly greater ECM-RRL than LbD, LbA + LbB and LbC + LbD (**Figure 2G**).

### Intraspecific Richness Effects on Productivity within Nutrient Treatments

There were no significant effects of *L. bicolor* intraspecific richness on the productivity of Scots pine and ECM fungi with either the inorganic (*P*-values < 0.091) or organic (*P*-values < 0.276) nutrient substrate (Supplementary Figure 1). Also, there were no strong associations (*R^2^*< 0.21) between productivity and ECM richness (Supplementary Figure 1).

### Transgressive Overyielding within Nutrient Treatments

**FIGURE 3:**
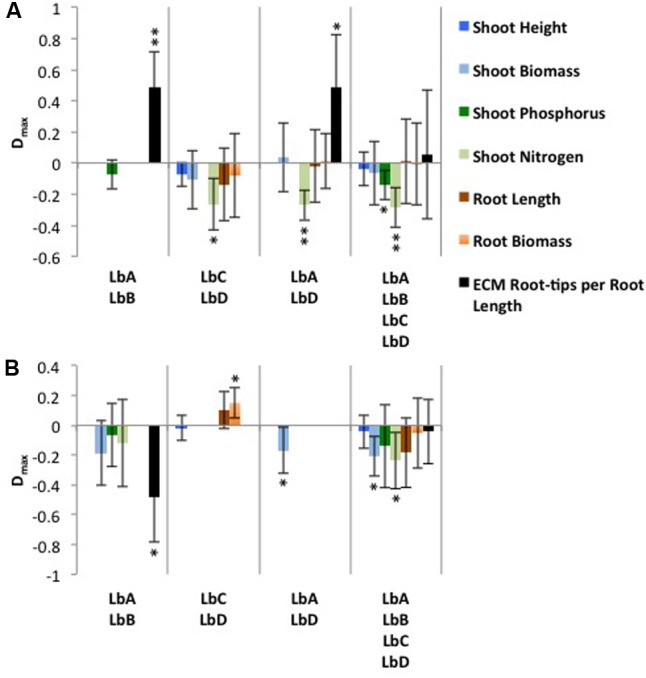
Transgressive overyielding (D_max_) of Scots pine plant and fungal productivity in response to *Laccaria bicolor* identity (LbA, LbB, LbC, LbD) under **(A)** inorganic and **(B)** organic nutrient substrates. A positive D_max_ value results when a mixture produces more than its most productive corresponding monoculture. Bars are absent for a productivity variable when the maximum yielding monoculture is not within a mixture. Mean ± SD is shown, and significance levels of the one sample *t*-test for difference from zero are indicated as: ^∗^*P* < 0.05 and ^∗∗^*P* < 0.01.

Under the inorganic nutrient treatment, the most productive *L. bicolor* monocultures were LbC for shoot height and LbD for shoot biomass, root biomass, root length and shoot N content, and LbA and LbB for ECM-RRL and shoot P content, respectively. For each mixture, the most productive corresponding monoculture means in calculations of transgressive overyielding were used, and resulted in mean *D*_max_ values ranging between -0.286 and 0.489, and with 26.3% of cases having positive *D*_max_ values (**Figure 3A**). In the mixtures LbA + LbB and LbA + LbD, Scot pine ECM-RRL was positively significantly different from zero, and thus indicating overyielding (**Figure 3A**).

Under the organic nutrient treatment, the most productive *L. bicolor* monocultures were LbC for shoot height, root biomass and root length, and LbA for shoot biomass, and LbB for shoot N and P content and ECM-RRL. For each mixture, the most productive corresponding monoculture means in calculations of transgressive overyielding were used, and resulted in mean *D*_max_ values ranging between -0.235 and 0.150, and with 13.3% of cases having positive *D*_max_ values (**Figure 3B**). In the mixture LbC + LbD, Scots pine root biomass was positively significantly different from zero, and thus indicating overyielding (**Figure 3B**).

### Mycorrhizal Colonization Effects on Plant Productivity

Comparison between the NM control and the *L. bicolor* monocultures for plant productivity under the inorganic nutrient substrate revealed some significant differences in Scots pine shoot height (*F* = 30.70; *P* < 0.001), shoot biomass (*F* = 8.29; *P* < 0.001) and shoot N content (*F* = 6.18; *P* = 0.001) (Supplementary Table 2). Under the organic nutrient substrate, Scots pine shoot height (*F* = 13.57; *P* < 0.001), shoot biomass (*F* = 3.31; *P* = 0.027) and shoot P content (*F* = 3.44; *P* = 0.023) were significantly different between the NM control and some of the *L. bicolor* monocultures (Supplementary Table 2). For both the inorganic and organic nutrient treatments, NM Scots pine were shortest and had the least biomass, but tended to contain more P and N in shoots and produced more roots compared to the *L. bicolor* monocultures (Supplementary Table 2).

## Discussion

In this study, we tested the effect of *L. bicolor* intraspecific identity and richness on the productivity of Scots pine and associated ECM fungal productivity in microcosms supplied with either an inorganic or organic composition of N and P nutrient resources. We predicted that identity and richness effects would be greatest when the nutrient resources in the substrate are chemically complex and diverse, due to the greater likelihood of niche partitioning (Turner, 2008). This prediction was supported by our observation of significant variation in growth of *L. bicolor* on a range of nutrient forms. When plants were grown in symbiosis, however, the experimental findings did not support our prediction. Intraspecific identity effects on plant productivity were only found when the Scots pine seedlings were grown in the inorganic nutrient substrate. Despite that intraspecific identity effects on fungal productivity were found under both the inorganic and organic nutrient substrate, the effects were not greater in response to the organic nutrient treatment. Furthermore, no effects of *L. bicolor* intraspecific richness on Scots pine plant and fungal productivity were found under either nutrient treatment.

The lack of richness and identity effects on productivity under the organic nutrient treatment reflects similar utilization of the nutrient resources rather than nutrient specialization by the *L. bicolor* strains. Our current understanding of intraspecific ECM variation in N source use is largely based on pure culture studies (Finlay et al., 1992; Anderson et al., 1999; Sawyer et al., 2003; Guidot et al., 2005), which can only serve as an estimate of the strains fundamental niche (Bogar and Peay, 2017). We demonstrated intraspecific variation in organic N source utilization by *L. bicolor* strains in pure culture, but these findings were not consistent with the effects on plant and fungal productivity when in symbiosis. For example, there were significant differences in mycelial biomass between the strains LbC and LbD when grown on media with glutamic acid and phenylalanine as the sole source of N, but not when grown on ammonium nitrate after 28 days. Furthermore, in comparison to ammonium nitrate, mycelial biomass was greater on glutamic acid and glycine. However, contrary to these findings, when in monoculture, Scots pine inoculated with monocultures of LbC and LbD did not differ in ECM-RRL in response to manipulation of nutrient resources, and when in mixture, additive effects were found under the inorganic nutrient substrate but not for the organic nutrient substrate. These contrasting findings could reflect the realized niches of the strains in regards to soil nutrient resources. Further examination of organic N source utilization and transfer to host plants, and competition for nutrient resources between strains of ECM species within soil would help to define realized niches.

It is, however, possible that free-living microbes could have invaded the initially sterile microcosm substrate and caused mineralization of the organic N and P forms and thereby releasing inorganic nutrient forms and reducing the potential for niche partitioning by the *L. bicolor* strains. Alternatively, nutrient complexity effects on intraspecific richness and identity for plant and fungal productivity may only occur if plants are nutrient starved. For example, it has been shown that when soil fertility is low the biomass of birch plants, originally associated with eight ECM species, doubled in comparison to single ECM associations (Jonsson et al., 2001). In our study, the mean shoot N and P concentrations (37 ± 9 mg N g^-1^; 9 ± 2 mg P g^-1^) of Scots pine relative to the optimal content for Scots pine needles (13–18 mg N g^-1^; 1.6–2.2 mg P g^-1^; Moilanen et al., 2010), suggest the plants were not nutrient limited. Soil nutrient availability is likely a strong influencing factor on complementarity and competition intensity among strains for nutrient resources (Jonsson et al., 2001).

Furthermore, the lack of intraspecific richness effects on plant and fungal productivity could be related to the number of available nutrient resources rather than the chemical form of the nutrients. In this study, the organic nutrient treatment was comprised of four N sources, whereas the inorganic nutrient treatment was comprised of two. Based on resource competition theory, the addition of a limiting resource (e.g., N from the addition of glutamic acid) making that resource (i.e., N) no longer limited could reduce the competitive trade-off of the strains (Tilman, 1982). Competitive trade-offs have recently been reported for ECM species at the root system scale (Moeller and Peay, 2016), and presumably competitive trade-offs could also occur between individuals within an ECM species. In this scenario, strain co-existence would be predicted to decrease with increasing resource number (Harpole et al., 2016). To test this hypothesis, ECM strain abundance data would be required, and an experimental design that disentangles nutrient complexity from resource number effects on productivity.

Although identity and richness effects of plant and fungal productivity were not greater in the organic nutrient treatment, the results do suggest that identity and richness effects on plant and fungal productivity are context dependant. In this study, *L. bicolor* identity effects on plant productivity were only found under the inorganic nutrient substrate, and although identity effects on fungal productivity were found for both nutrient complexities, different trends in productivity emerged between the nutrient treatments. For example, under the inorganic nutrient substrate, Scots pine ECM-RRL was greater in the *L. bicolor* mixtures than the monocultures, and transgressive overyielding occurred, suggesting a degree of complementarity. In the organic nutrient substrate, Scots pine ECM-RRL varied within and amongst the *L. bicolor* mixtures and monocultures. This finding possibly reflects variation in nutrient source utilization by the *L. bicolor* strains, but also that competition for a nutrient resource could be occurring in some of the mixtures.

No significant richness effects on plant and fungal productivity were found, which contrasts with past work (Hazard et al., 2017). Hazard et al. (2017) used the same *L. bicolor* strains, similar experimental design but with a different, single growth medium and lower concentration of the inorganic nutrient solution, and found that intraspecific richness increased Scots pine root productivity and ECM-RRL. In the present study, significant transgressive overyielding of ECM-RRL occurred in the mixtures that contained two *L. bicolor* strains, and there was a weak trend, of increasing ECM-RRL with increasing intraspecific *L. bicolor* richness under the inorganic nutrient treatment. Conversely, *L. bicolor* identity affected plant and fungal productivity, an effect also seen by Hazard et al. (2017), but in the present study, the identity effects were stronger. Shoot height, shoot biomass and root length of Scots pine were significantly greater under the organic nutrient treatment for some of the *L. bicolor* strains, mostly for the monocultures, whereas shoot P content of Scots pine was greater under the inorganic nutrient treatment. The difference in the strength of the richness and identity relationships with plant and fungal productivity found between these studies might reflect the different growth media, i.e., peat-vermiculite versus sand-vermiculite, and the difference in nutrient availability. Peat-vermiculite may support more physical niches and diverse forms of nutrients, due to the inherit differences in water holding capacity, aeration, bulk density and cation exchange capacity (Sahin et al., 2002). Also, a lower nutrient rich substrate may enhance resource complementarity (Jonsson et al., 2001) between the *L. bicolor* strains. Collectively, these findings suggest that substrate physical and chemical conditions interact with ECM fungal richness and identity to affect plant and fungal productivity metrics. Also, these findings suggest that the substrate properties are driving the development of certain functional traits by interacting with particular *L. bicolor* strains. Further studies conducted with other ECM species and strains within a species, and under more natural nutrient conditions and across a range of forest soil types would be valuable for identifying patterns for ECM diversity effects on plant and fungal productivity.

## Conclusion

Intraspecific variation in *L. bicolor* nutrient resource utilization did not interact with substrate nutrient complexity when the fungi were grown in symbiosis with Scots pine. As similar levels of intraspecific variation in nutrient utilization have been observed with other ECM species (e.g., *Amanita muscaria*, Sawyer et al., 2003; *Hebeloma cylindrosporum*, Guidot et al., 2005), our findings suggest that ECM intraspecific richness may be important for plant and fungal productivity under nutrient limiting conditions, relative to resource heterogeneity under higher resource conditions. Our data suggests intraspecific identity of ECM fungi becomes more important than richness in modulating plant and fungal performance under nutrient rich conditions.

## Author Contributions

CH, AT, and DJ planned and designed the research. CH performed research and data analysis. CH, LK, HD, and FM collected data. CH and DJ wrote the manuscript.

## Conflict of Interest Statement

The authors declare that the research was conducted in the absence of any commercial or financial relationships that could be construed as a potential conflict of interest.
