# Supplementary figures and images for: Strain Identity of the Ectomycorrhizal Fungus Laccaria bicolor Is More Important than Richness in Regulating Plant and Fungal Performance under Nutrient Rich Conditions
Source: Front Microbiol. 2017 Sep 26;8:1874. doi: 10.3389/fmicb.2017.01874 (PMC5622926; doi:10.3389/fmicb.2017.01874)

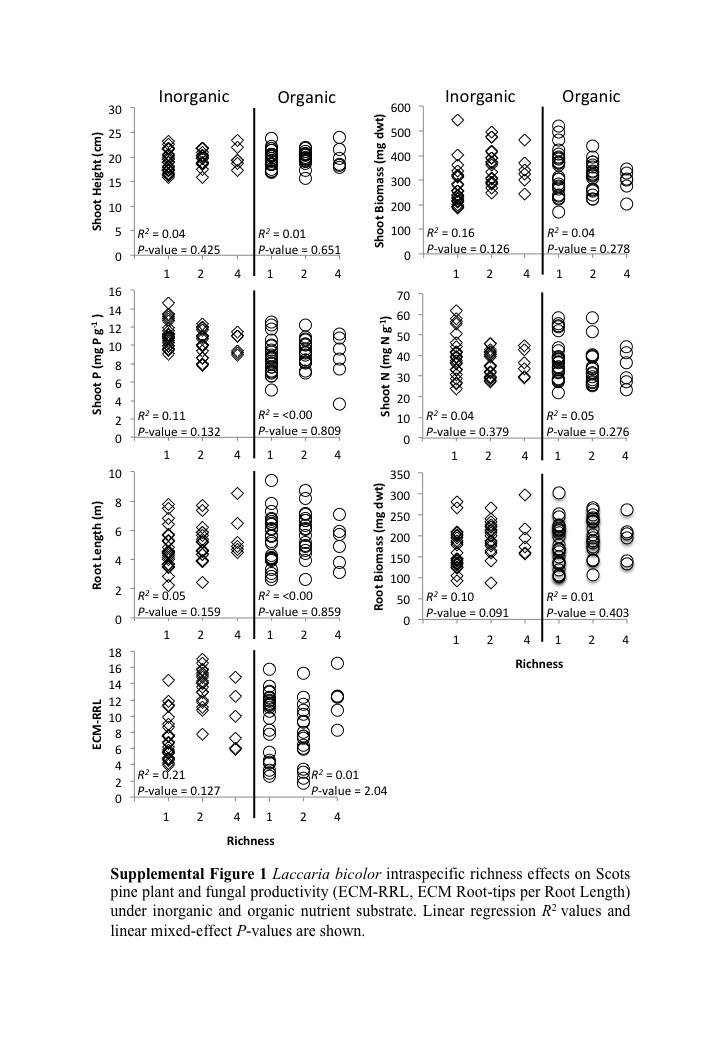

Supplement: Supplementary file 1 [file Image_1.JPEG]
