# Supplementary material for: Strain Identity of the Ectomycorrhizal Fungus Laccaria bicolor Is More Important than Richness in Regulating Plant and Fungal Performance under Nutrient Rich Conditions
Source: Front Microbiol. 2017 Sep 26;8:1874. doi: 10.3389/fmicb.2017.01874 (PMC5622926; doi:10.3389/fmicb.2017.01874)
Supplement: Supplementary file 2 [file Table_1.PDF]

Supplemental Table 1 Mean plant and fungal measures of seedlings planted in each of the experimental microcosm treatments. *P*-values are derived from one-way analysis of variance, and *P*-values in parentheses were derived from including the non-mycorrhizal control. Means (±SD) with different letters are significantly different based on Tukey's *post hoc* test.

	**Seedling Biomass (mg FW)**			**Root Length (cm)**			**ECM root-tips per root length**		
**Strain Identity**	Mean	SD	*P*	Mean	SD	*P*	Mean	SD	*P*
**Inorganic Nutrient Substrate**			0.092 (0.001)			0.244 (0.001)			0.080
LbA	0.406	0.032		47.4	7.1		4.18	1.33	
LbB	0.406	0.064		44.1	9.8		6.20	2.93	
LbC	0.437	0.128		39.2	17.8		4.17	2.43	
LbD	0.310	0.021		36.2	9.0		3.44	1.50	
LbA + LbB	0.429	0.060		50.0	10.1		4.17	1.40	
LbC + LbD	0.346	0.063		37.4	9.2		3.58	0.76	
LbA + LbD	0.361	0.056		42.6	10.0		3.14	0.56	
LbA + LbB + LbC + LbD	0.375	0.080		39.0	2.6		3.66	0.66	
Non-mycorrhizal control	0.210	0.032		13.9	2.0		-	-	
**Organic Nutrient Substrate**			1.000 (0.282)			0.148 (0.001)			0.834
LbA	0.377	0.050		58.3^{a}	16.4		3.72	1.72	
LbB	0.368	0.044		49.0^{a}	7.0		4.62	1.08	
LbC	0.364	0.097		43.5^{a}	21.5		4.48	1.87	
LbD	0.383	0.028		37.8^{ab}	8.7		3.50	1.46	
LbA + LbB	0.384	0.037		49.5^{a}	9.5		4.19	1.79	
LbC + LbD	0.389	0.074		44.9^{a}	16.6		3.70	1.06	
LbA + LbD	0.360	0.055		43.8^{a}	6.5		3.67	1.24	
LbA + LbB + LbC + LbD	0.370	0.064		37.4^{ab}	8.7		3.96	0.73	
Non-mycorrhizal control	0.212	0.011		16.3^{b}	2.6		-	-	
