# Supplementary material for: Strain Identity of the Ectomycorrhizal Fungus Laccaria bicolor Is More Important than Richness in Regulating Plant and Fungal Performance under Nutrient Rich Conditions
Source: Front Microbiol. 2017 Sep 26;8:1874. doi: 10.3389/fmicb.2017.01874 (PMC5622926; doi:10.3389/fmicb.2017.01874)
Supplement: Supplementary file 3 [file Table_2.PDF]

Supplemental Table 2 Comparison between *Laccaria bicolor* (Lb) monocultures and non-mycorrhizal (NM) control for plant productivity under (a) inorganic and (b) organic nutrient substrate. Means (±SD) with different letters are significantly different based on Tukey's *post hoc* test following one-way analysis of variance (*P*-values < 0.05 are in bold).

	**Shoot Height (cm)**			**Shoot Biomass (mg dwt)**			**Shoot P (mg P g^{-1})**			**Shoot N (mg N g^{-1})**		
	Mean	SD	*P*	Mean	SD	*P*	Mean	SD	*P*	Mean	SD	*P*
**(a) Inorganic**			**<0.001**			**<0.001**			0.084			**0.001**
LbA	20.3^{a}	2.1		232^{b}	46.1		11.1	1.0		35.8^{c}	6.1	
LbB	17.4^{b}	1.1		222^{b}	49.5		11.8	1.6		35.1^{c}	7.1	
LbC	20.7^{a}	1.1		264^{b}	29.8		11.3	1.7		38.6^{bc}	6.9	
LbD	17.2^{b}	0.6		364^{a}	103.4		10.5	1.3		50.6^{ab}	13.0	
NM control	13.1^{c}	0.4		176^{b}	13.4		13.0	1.0		53.7^{a}	5.4	
**(b) Organic**			**<0.001**			**0.027**			**0.023**			0.144
LbA	19.9^{a}	1.6		370^{a}	100		9.0^{ab}	1.9		40.3	7.4	
LbB	20.1^{a}	1.3		328^{ab}	73		9.9^{ab}	1.6		44.1	9.6	
LbC	20.2^{a}	2.1		368^{ab}	89		7.3^{b}	1.3		31.7	6.0	
LbD	17.7^{a}	1.1		274^{ab}	84		8.5^{ab}	1.6		38.6	13.3	
NM control	14.4^{b}	1.3		222^{b}	49		10.4^{a}	0.9		46.6	11.7	

	**Root Length (m)**			**Root Biomass (mg dwt)**		
	Mean	SD	*P*	Mean	SD	*P*
**(a) Inorganic**			0.235			0.149
LbA	4.62	1.24		143	35	
LbB	4.03	1.02		143	38	
LbC	4.54	0.84		169	29	
LbD	5.64	1.96		201	64	
NM control	5.67	1.78		155	39	
**(b) Organic**			0.078			0.731
LbA	4.60	1.56		169	55	
LbB	4.26	1.27		165	47	
LbC	6.24	1.40		201	36	
LbD	6.16	1.94		189	71	
NM control	7.24	3.03		203	82	
