# Supplementary material for: Strain Identity of the Ectomycorrhizal Fungus Laccaria bicolor Is More Important than Richness in Regulating Plant and Fungal Performance under Nutrient Rich Conditions
Source: Front Microbiol. 2017 Sep 26;8:1874. doi: 10.3389/fmicb.2017.01874 (PMC5622926; doi:10.3389/fmicb.2017.01874)
Supplement: Supplementary file 4 [file Table_3.PDF]

Supplemental Table 3 Summary and model statistics for overall main and interactive effects for nutrient treatments and intraspecific richness on Scots pine plant and fungal productivity.

Productivity Variable	Factor	Summary Statistics		Model Statistics		
		Mean	SD	df	*T*-value	*P*-value
Shoot Height (cm)	Nutrient Treatments			11	0.580	0.573
	Inorganic	19.34	1.96			
	Organic	19.66	1.75			
	Richness			1	0.687	0.616
	1	19.22	1.96			
	2	19.73	1.56			
	4	19.97	2.19			
	Nutr. Treat.*Richness			11	-0.401	0.695
Shoot Biomass (mg dwt)	Nutrient Treatments			11	0.141	0.560
	Inorganic	313.31	88.3			
	Organic	323.17	76.8			
	Richness			1	2.280	0.263
	1	303.32	91.77			
	2	338.60	71.20			
	4	316.85	65.38			
	Nutr. Treat.*Richness			11	-2.147	0.054
Shoot Phosphorus (mg P g^{-1})	Nutrient Treatments			11	-2.937	**0.013**
	Inorganic	10.67	1.50			
	Organic	8.91	1.80			
	Richness			1	-1.618	0.352
	1	9.97	2.07			
	2	9.71	1.46			
	4	9.35	2.18			
	Nutr. Treat.*Richness			11	1.371	0.197
Shoot Nitrogen (mg N g^{-1})	Nutrient Treatments			11	-0.159	0.876
	Inorganic	38.08	8.69			
	Organic	36.52	9.51			
	Richness			1	-0.388	0.763
	1	39.35	10.11			
	2	35.37	7.70			
	4	34.95	7.25			
	Nutr. Treat.*Richness			11	-0.091	0.928
Root Length (m)	Nutrient Treatments			11	1.139	0.278
	Inorganic	496.49	137.4			
	Organic	550.81	161.5			
	Richness			1	0.914	0.528
	1	501.42	157.61			
	2	548.03	144.95			
	4	539.46	145.89			
	Nutr. Treat.*Richness			11	-0.642	0.533
Root Biomass (mg dwt)	Nutrient Treatments			11	1.141	0.277
	Inorganic	180.65	48.2			
	Organic	190.32	49.4			
	Richness			1	1.357	0.404
	1	172.75	50.49			
	2	199.12	42.90			
	4	195.55	48.56			
	Nutr. Treat.*Richness			11	-0.840	0.418

Supplemental Table 3 Continued.

Productivity Variable	Factor	Summary Statistics		Model Statistics		
		Mean	SD	df	*T*-value	*P*-value
ECM-Root Tips per Root Length	Nutrient Treatments			11	0.624	0.544
	Inorganic	9.92	3.98			
	Organic	9.03	3.89			
	Richness			1	1.303	0.416
	1	9.97	2.07			
	2	10.62	4.26			
	4	10.74	3.38			
	Nutr. Treat.*Richness			11	-0.921	0.376
